# Senescence-Associated Pro-inflammatory Cytokines and Tumor Cell Plasticity

**DOI:** 10.3389/fmolb.2020.00063

**Published:** 2020-05-13

**Authors:** Jean Paul Vernot

**Affiliations:** Facultad de Medicina, Universidad Nacional de Colombia, Bogotá, Colombia

**Keywords:** Plasticity, senescence, proinflammatory cytokines, EMT, hybrid phenotypes, CSC

## Abstract

The well-recognized cell phenotypic heterogeneity in tumors is a great challenge for cancer treatment. Dynamic interconversion and movement within a spectrum of different cell phenotypes (cellular plasticity) with the acquisition of specific cell functions is a fascinating biological puzzle, that represent an additional difficulty for cancer treatment and novel therapies development. The understanding of the molecular mechanisms responsible for moving or stabilizing tumor cells within this spectrum of variable states constitutes a valuable tool to overcome these challenges. In particular, cell transitions between epithelial and mesenchymal phenotypes (EMT-MET) and de-and *trans*-differentiation processes are relevant, since it has been shown that they confer invasiveness, drug resistance, and metastatic ability, due to the simultaneous acquisition of stem-like cell properties. Multiple drivers participate in these cell conversions events. In particular, cellular senescence and senescence-associated soluble factors have been shown to unveil stem-like cell properties and cell plasticity. By modulating gradually the composition of their secretome and the time of exposure, senescent cells may have differential effect not only on tumor cells but also on surrounding cells. Intriguingly, tumor cells that scape from senescence acquire stem-like cell properties and aggressiveness. The reinforcement of senescence and inflammation by soluble factors and the participation of immune cells may provide a dynamic milieu having varied effects on cell transitions, reprogramming, plasticity, stemness and therefore heterogeneity. This will confer different epithelial/mesenchymal traits (hybrid phenotype) and stem-like cell properties, combinations of which, in a particular cell context, could be responsible for different cellular functions during cancer progression (survival, migration, invasion, colonization or proliferation). Additionally, cooperative behavior between cell subpopulations with different phenotypes/stemness functions could also modulate their cellular plasticity. Here, we will discuss the role of senescence and senescence-associated pro-inflammatory cytokines on the induction of cellular plasticity, their effect role in establishing particular states within this spectrum of cell phenotypes and how this is accompanied by stem-like cell properties that, as the epithelial transitions, may also have a continuum of characteristics providing tumor cells with functional adaptability specifically useful in the different stages of carcinogenesis.

## Cell Heterogeneity and Cancer Stem Cell Plasticity

Phenotypic and functional intratumour heterogeneity occurs in cancer cells as a consequence of the clonal evolution and cancer stem cell models, genetic and epigenetic alterations, microenvironment cues, and importantly, reversible and dynamic modifications in cellular properties (Marjanovic et al., 2013; Meacham and Morrison, 2013; Mooney et al., 2016; Gupta et al., 2019). The high degree of genetic heterogeneity fuelled in part by genetic instability and clonal evolution is responsible for a good part of this phenotypic heterogeneity during tumor development (Loeb and Monnat, 2008; Mimori et al., 2018; Turajlic et al., 2019). Dynamic conversion between phenotypic states, bidirectional processes of differentiation and de-differentiation of cancer stem cells (CSC), contributes particularly to this heterogeneity and allows functional adaptation of cancer cells to the different hurdles imposed by the homeostatic and cell growth control processes (ElShamy and Duhé, 2013; Marjanovic et al., 2013; Nieto et al., 2016). An effective cancer treatment is enormously limited by this phenotypic heterogeneity and cellular plasticity, and particularly by the lack of understanding of the specific contribution of the different molecular and cellular mechanisms used for their establishment (Marusyk et al., 2012).

It is also recognized that cellular heterogeneity can also be found in other components of the tumor microenvironment (TME). Early studies have revealed distinct functional roles of the different stromal elements with the capacity to define a benign or transformed cell phenotype (Shekhar et al., 2001). Stroma-mediated epigenetic cues generate a permissive TME that may reverse breast cancer cell limitations and promote tumor cell plasticity and growth (Varga et al., 2014; Haynes et al., 2017).

On the other hand, it is now well recognized that cell identity is more ambiguous and cell fate less predictable as previously thought (Mills et al., 2019), and that in pathological conditions these features are much more complex. In fact, cell identity becomes more plastic and cell fate more uncertain when homeostasis of the microenvironment is perturbed, e.g., during injury, senescence, inflammation or cancer (Lamouille et al., 2014). Principally, two mechanisms responsible of tumor cell plasticity contributing not only to tumor initiation and progression (Varga et al., 2014) but also to chemoresistance and cancer relapse (Le Magnen et al., 2018) have been identified: the epithelial-mesenchymal transition (EMT) and de-differentiation or *trans*-differentiation processes. In this review, we discuss how senescence affect tumor cell plasticity through these mechanisms, generating tumor cells with particular stemness features that allow their functional adaptation during cancer progression. A particular emphasis is made on pro-inflammatory cytokines (IL-6 and IL-8) secreted by senescent/inflammatory cells, known to induce EMT, cell migration, and de-differentiation, and be responsible for propagating senescence and inflammation in the TME.

## EMT/CSC and Tumor Cell Plasticity

One of the most well known examples of cellular plasticity is the epithelial-to-mesenchymal transition (EMT), which occurs in several contexts in normal development and disease, including cancer (Oft et al., 1996; Thiery, 2002; Kalluri and Weinberg, 2009; Thiery et al., 2009). The EMT has been associated with the appearance of cancer cells with stem-like cell properties or CSC (Mani et al., 2008; Morel et al., 2008) which are thought to be responsible for driving cancer growth, inducing radio- and chemotherapy resistance, causing metastasis and relapse (Clarke et al., 2006). The EMT can be induced by several of numerous pleiotropic growth factors or cytokines (EGF, HGF, FGF, TGF-β, NOTCH, IL-6, IL-8, among others) (Thiery et al., 2009; Kim et al., 2016) that can be produced by different cells present in the tumor. This leads to the redundant expression of the EMT-associated transcription factors (EMT-TF), including SNAIL1/2, TWIST1/2, ZEB1/2, and PRRX (Nieto et al., 2016; Yuan et al., 2019), and subsequent epithelial genes repression and mesenchymal genes induction (Lamouille et al., 2014; Dongre and Weinberg, 2019).

Intriguingly, a CSC signature could include epithelial gene expression within a mesenchymal cell context, or mesenchymal gene expression within a background of mostly epithelial tumor cells (Grosse-Wilde et al., 2015). In fact, CSC may exist in distinct mesenchymal- and epithelial-like states and possess a high degree of cellular plasticity enabling cells to dynamically transit between these two states based mainly on signals they receive from the TME (Liu et al., 2014). Cells displaying these dynamic changes have been called, plastic or metastable cells, cells in transition states, etc. (Mills et al., 2019). Therefore, cellular plasticity of tumor cells has been also associated with the reverse program MET (together with the EMT, better called epithelial-mesenchymal plasticity –EMP) (Guarino et al., 1999; Ford and Thompson, 2010; Lu et al., 2013; Nieto, 2013; Luo et al., 2015) and with the existence of the hybrid (E/M) phenotype (Christiansen and Rajasekaran, 2006; Grosse-Wilde et al., 2015; Jolly et al., 2015a). The E/M phenotype may appear in a fine-tuned manner allowing collective migration of cohesive epithelia (Revenu and Gilmour, 2009) and having multiple benefits that can be exploited by tumor cells during the different stages of cancer progression (Friedl et al., 2012; Jolly et al., 2015a). Since the hybrid phenotype may exist in different ranges between the pure E and M stages, it is particularly relevant that more cellular plasticity and tumorigenicity has been found in hybrid cells that tend to be more epithelial (Ocaña et al., 2012; Patsialou et al., 2012; Ombrato and Malanchi, 2014; Grosse-Wilde et al., 2015). A strong association between the expression of genes responsible for this hybrid E/M phenotype and breast cancer cell invasive behavior and aggressiveness has been found (Hendrix et al., 1997; Livasy et al., 2006; Jolly et al., 2015a). In a TNBC model it was also found that CSC cells residing in an intermediate E/M phenotypic state had more stem-like cell properties, tumor-initiating capability and worse prognosis (Bierie et al., 2017). Although, the hybrid E/M phenotype has been considered metastable, stability factors of the hybrid E/M state have been described (Jia et al., 2015; Jolly et al., 2016). Intriguingly, stability was accompanied by a gain of stemness functions (Jolly et al., 2015b). Very recently, highly tumourigenic breast cancer cells in a stable intermediate E/M phenotype, driven by the mesenchymal SNAIL TF and the stemness-associated Wnt signaling pathway were more tumourigenic than a mixture of cells at the end of the spectrum of E or M phenotypes (Kröger et al., 2019). Nevertheless, the use of additional markers could eventually discern cell subpopulations of CSC within this E/M stable phenotype. Indeed, in another study using three different cell surface markers it was possible to distinguish different intermediate states, that were functionally distinct in invasion, clonogenicity, differentiation and importantly, plastic cell properties (Pastushenko et al., 2018).

Paradoxically, the EMT phenotype has also been shown to prevent the acquisition of stem-like cell properties (Korpal et al., 2011; Celià-Terrassa et al., 2012; Sarrio et al., 2012). Also, cooperation between EMT and non-EMT cells could favor lung metastasis by the non-EMT cells (Tsuji et al., 2008). More recently, in an *in vivo* breast cancer model it was shown that cells in a mesenchymal phenotype arriving at a secondary site adopt an epithelial state after very few cell divisions (Beerling et al., 2016), suggesting that differences in stemness between epithelial and mesenchymal states could be lost under certain circumstances and become irrelevant for metastatic outgrowth.

**FIGURE 1:**
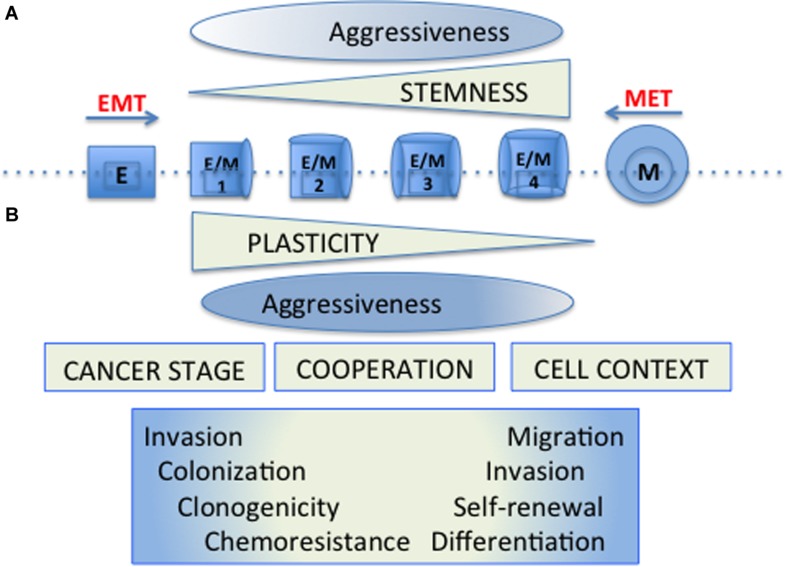
Cellular plasticity and tumor cell aggressiveness. **(A)** The original model of cellular plasticity proposed that the EMT program was associated with the appearance of CSC properties and it was supposed that these mesenchymal-like cells were responsible for driving cancer cell growth, chemotherapy resistance, metastasis and relapse. **(B)** The new models of cellular plasticity propose that stemness properties would be of different functional significance depending on the cell context and the particular cancer progression stage. In these models, hybrid cells (E/M1-4) expressing both epithelial (E) and Mesenchymal (M) markers develop cellular plasticity with different stemness properties, allowing tumor cells to adapt to the diverse circumstances of the tumor microenvironment, and to take special advantages of the functional properties conferred by the establishment of these programs. The aggressiveness would be manifested by a wide spectrum of distinct hybrid cells, requiring particular properties according to the hurdles present during tumourigenesis.

Thus the EMT and CSC programs that were originally described as coincident may appear separately, be regulated differentially and have gradual “intensities” (Beck et al., 2015; Schmidt et al., 2015; Batlle and Clevers, 2017). Stemness properties would be of different functional significance depending on the cell context and the cancer progression stage. In these scenarios, cellular plasticity allows tumor cells to adapt to the different circumstances of the tumor microenvironment, and to take advantage differentially of the functional properties conferred by the establishment of these programs (Figure 1).

### Role of Senescence and Inflammation in EMP and CSC Properties Acquisition

**FIGURE 2:**
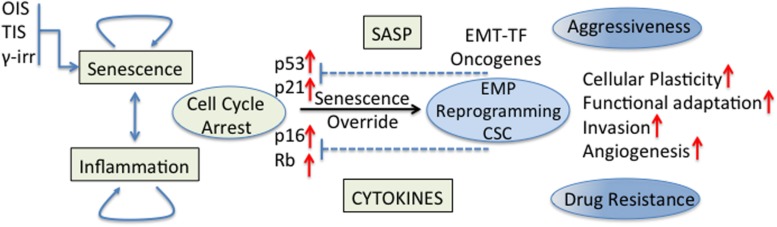
Senescence/inflammation and cellular plasticity. Tumor cell senescence override is necessary for full malignancy. EMT-TF cooperate with oncogenic signals to abrogate key regulators of cell cycle for a complete induction of the EMT program and the acquisition of stemness properties. Some EMT-TF induce cellular plasticity and drug resistance through regulation of signaling pathways (NF-kB and MAPK) involved in stem cell maintenance. The final effect of senescence/inflammation in EMT/CSC plasticity depends on the contextual signals present in the TME, the stage of cancer progression and the functional heterogeneity reached.

The effect of senescent and inflammatory phenomena occurring in the TME on cellular plasticity and tumor progression is of great interest in cancer biology. Cellular senescence was recognized as a powerful anti-cancer mechanism (Campisi, 2005) since stressed or damaged cells are permanently withdrawn from the cell cycle. Nevertheless, early work has also shown that cancer cells can evade this tumor suppressive mechanism in different ways, for example, the p16 inactivation by CpG island methylation (Foster et al., 1998); this being just one of other evasion mechanisms that began to be revealed decades ago (Hollstein et al., 1991; Kim et al., 1994; Shay and Bacchetti, 1997; Jarrard et al., 1999). Currently, it is believed that cancer cell senescence override is necessary for full malignancy (Collado et al., 2005; Ohashi et al., 2010). Indeed, it was shown that human cancers express EMT-TF that are able to abrogate key regulators of senescence (for example, p53 and Rb) and cooperate with oncogenic signals allowing the complete induction of an EMT program and the acquisition of invasiveness properties (Ansieau et al., 2008; Tran et al., 2012). Some EMT-TF could also induce cellular plasticity and drug resistance through regulation of signaling pathways (NF-kB and MAPK) involved in stem cell maintenance (Lim et al., 2013; Figure 2). Also in an experimental model of TNBC, p53 deletion from the mammary epithelium inhibited the expression of differentiation markers, induced an early expansion of mammary stem/progenitor cells and accelerated the formation of TNBC tumors (Chiche et al., 2017).

Not only intrinsic senescence in the tumor cells and their scape from this condition is relevant for disease progression. Changes in the supportive stroma could also affect growth and homeostasis of tissues and be responsible for cancer progression and aggressiveness. Senescent cells induced by irradiation, drug treatment, oncogenic stimuli and other stressful insults can also exert detrimental effects on cancer cells and the surrounding tissues. The tumor stroma consists of non-malignant cells, including resident cells such as cancer-associate fibroblasts (CAF), endothelial cells and pericytes, immune cells, and mesenchymal stroma cells, among others (Eiro et al., 2019). All these cells can impact tumor growth in different modes. CAF, for example, can induce tumourigenesis of epithelial cells (Olumi et al., 1999; Shimoda et al., 2010). Additionally, because CSC can differentiate into supportive CAF-like cells they are able to induce and ensure, by this mean, survival of cancer cells in the TME (Nair et al., 2017). Tumour-associated macrophages (TAM) are the most abundant infiltrated leukocyte in the TME, in general they are polarized to the M2 phenotype and secrete IL-8, showing either anti- or pro-tumourigenic effects (Mantovani, 2004; Sica et al., 2006; Dominguez et al., 2017). TAM infiltration has a positive correlation with IL-8 expression and a negative correlation with patient survival (Chen et al., 2003). Although senescence-associated fibroblasts vary in different ways from CAF, they are also able to promote the proliferation and tumourigenesis of pre-neoplastic or neoplastic epithelial cells (Krtolica et al., 2001; Coppé et al., 2010).

Interestingly, these effects were independent of the senescence inducer and mainly due to factors secreted by senescent fibroblasts (cytokines, chemokines, growth factors and proteases) (Krtolica et al., 2001), collectively known as the senescence-associated secretory phenotype (SASP) (Coppé et al., 2008). The SASP was able to alter epithelial differentiation and to induce EMT (Parrinello et al., 2005; Bavik et al., 2006; Coppé et al., 2008). High levels of the pro-inflammatory cytokines IL-6 and IL-8, the main soluble factors present in the SASP, are responsible for augmenting the invasiveness of a panel of breast cancer cell lines (Coppé et al., 2008). Not surprisingly IL-6 and IL-8, having a central role in CSC formation (Korkaya et al., 2011) were highly upregulated in response to IL-1-beta (Lim et al., 2013) and responsible for SASP propagation in the TME (Coppé et al., 2010). The secretion of matrix metalloproteinases by senescent cells also promotes the invasion of cancer cells (Parrinello et al., 2005; Liu and Hornsby, 2007).

Likewise, inflammation, an important physiological process in the TME can, on one hand, induce cell proliferation and survival of cancer cells, and promote angiogenesis (Mantovani et al., 2008) and on the other, attract immune cells, in particular TAM a key cell subset contributing to inflammation and resulting in anti-tumor activity (De Visser et al., 2006; Sica et al., 2006; Shigdar et al., 2014). These effects are also mediated by inflammatory cytokines (IL-1, IL-6, IL-8, TNF, TGF-ß, and others) and chemokines (CCL2 and CXCL8, for example) especially having an important effect during chronic inflammation (Tanno and Matsui, 2011). In particular, the EMT-inducing effect of TGF-ß has been well recognized (Hollier et al., 2009). IL-6 regulates the self-renewal of breast CSC through activation of STAT3 and NF-kB and further inducing an even greater secretion of IL-6 and IL-8 (Bromberg and Wang, 2009). IL-8 also increases breast CSC self-renewal by increasing the expression of the receptor CXCR1 (Ginestier et al., 2010). Elevated serum levels of both cytokines have been used independently as prognostic markers for breast cancer (Bachelot et al., 2003; Benoy et al., 2004; Knüpfer and Preiß, 2007; Yao et al., 2007).

It is believed, that this senescent/inflammatory milieu contains the main contextual signals present in the TME, with the capability to affect pre- and malignant cells. In the post-crisis immortalized HEK cells model (Castro-Vega et al., 2013), cells with chromosomal instability, initially unable to induce tumors on their own in spite of displaying an EMT phenotype, become fully tumorigenic only in the presence of senescent fibroblasts (Castro-Vega et al., 2015). The acquisition of this tumor capacity was accompanied by the presence of enhanced stem-like cell properties. Interestingly, cells recovered from those tumors were now endowed with cell-autonomous tumorigenicity (in the absence of senescent cells), but they exhibited extensive heterogeneity in cell phenotype, differentiation status, gene expression profile and response to SASP (Castro-Vega et al., 2015). The capacity of SASP to modulate these phenotypes/functions (phenotypic interconversions, functional plasticity and adaptation) in pre- or fully malignant cells in the different stages of cancer progression must be very relevant *in vivo* (Chaffer and Weinberg, 2015).

From the known SASP composition and the inflammatory mediators it is clear that there is a close relationship between senescence and inflammation (Lasry and Ben-Neriah, 2015) and a strong effect of both processes in tumor progression. Prolonged exposure to inflammatory mediators can enhance tumor growth (Krtolica et al., 2001; Bavik et al., 2006). Also, chronic inflammation increased cancer risk by promoting tumourigenesis (Balkwill et al., 2005). The fact that the use of non-steroidal anti-inflammatory drugs after surgical removal of tumors decreases relapse and mortality in different types of cancer, suggests an important and broad role of inflammation in tumor progression (Fraser et al., 2014; Streicher et al., 2014). Due to their dual effect in cancer suppression and promotion, and their reciprocal influence, the final effect of senescence/inflammation in EMT/CSC plasticity would depend on the stage of cancer progression, the contextual signals present in the TME, the capacity of the tumor cells to escape senescence and the functional heterogeneity achieved (differentiation state, stemness attributes). Additionally, recent evidence supports the view that senescent and inflammatory mediators destabilize cancer cell genome, contributing to the accumulation of random genetic alterations and to the establishment of a genomically heterogeneous tumor cell population that can be further selected according to their new physiological properties and their relevance in the different stages of carcinogenesis (Colotta et al., 2009; Anuja et al., 2017).

## De- and *Trans*-Differentiation Processes and Cellular Plasticity Acquisition

De- and *trans*-differentiation processes, by which cells adopt a different differentiation state from the original one or a more primitive state, influence cellular plasticity and cell fate options. Recent studies in breast cancer recognize a connection between genetics/epigenetics alterations (oncogenes, tumor suppressor genes or cell lineage specifiers) and the cell of origin, either luminal or basal subtypes (Chu et al., 2019). These alterations in a particular cell context can induce cell reprogramming in lineage-committed mammary epithelial cells. Based on this observation and the relationship between induced pluripotent stem cells and cancer cells, it was proposed that one or more reprogramming factors could be involved in the spontaneous de-differentiation and the acquisition of stemness function (Leis et al., 2012).

For example, over-expression of SOX2 increased both the amount of mammospheres in an *in vitro* culture model in low binding conditions and the formation of tumors in a xenograft model. It was argued that reactivation of SOX2 could explain tumor heterogeneity by placing the self-renewal ability and tumor-initiating capacity in any cell along the axis of mammary differentiation (Leis et al., 2012). Also, in the immortalized non-tumorigenic MCF10A mammary epithelial cell line, the introduction of reprogramming factors (SOX2, KLF4, OCT4, and c-Myc) endowed MCF10A cells with CSC properties and malignant traits both *in vitro* and *in vivo* (Nishi et al., 2014). Likewise, the expression of oncogenic PIK3CA in uni-potent basal cells gave rise to luminal-like cells, while its expression in uni-potent luminal cells produced basal-like cells, before progressing into invasive tumors with heterogeneous breast cancer cell types (Koren et al., 2015; Van Keymeulen et al., 2015). Additionally, c-Myc, part of the Yamanaka reprogramming factors (Takahashi et al., 2007) and a downstream effector of PIM1 and IL-6 stimulation (Gao et al., 2019) induced mammary tumourigenesis through cell reprogramming, which was attributed to MYC-mediated repression of luminal fate-specific enhancers (Poli et al., 2018a). On the other hand, MYC-driven de-differentiation induced a stem-like cell state with activation of *de novo* enhancers, driving the transcriptional activation of oncogenic pathways (Poli et al., 2018a). These studies and others (reviewed in Chu et al., 2019) have allowed unveiling the connections between the reprogramming of committed mammary epithelial cells and the tumorigenic capabilities of heterogeneous breast cancer cells (Dravis et al., 2018; Rodilla and Fre, 2018).

In breast cancer it is thought that cancer cells are derived from a common luminal progenitor cell type with stem-like properties that is able to develop into both luminal and basal tumors (Granit et al., 2014). The existence of unrestricted gene expression patterns in this bi-potential progenitor cells represents the developmental biology support for the occurrence of the hybrid phenotype (Fillmore and Kuperwasser, 2008). The hybrid signature was associated with stemness functions and with a poorest outcome in all breast cancer subtypes (luminal and basal breast cancer patients) (Grosse-Wilde et al., 2015). This strong relationship is based on the stemness appeal of the hybrid phenotype based on the cooperation between the E and M states complementing their functional attributes, cellular plasticity conveyed by the E state and self-renewal carried out by the M state (Grosse-Wilde et al., 2015). Also, in a transforming model of basal-like breast cancer it was shown that differentiated cells could give rise to CSC *in vitro* and *in vivo* (Chaffer and Weinberg, 2011). The authors argued that the plasticity of CSC could be associated with the cell characteristics in their respective non-stem cell state and their capacity to produce new CSC.

### Cellular Senescence and Cell Reprogramming

**FIGURE 3:**
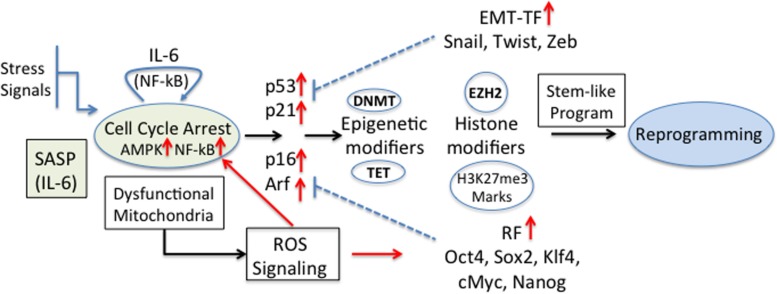
Senescence and tumourigenic cell reprogramming. Different stress stimuli induce cellular senescence and cell cycle arrest with a concomitant expression of the regulators [p53, p21CIP1 (p21), p16INK4a (p16), p14Arf (p14)] and induction of SASP. In particular IL-6, being one of the main components within SASP, is a key player in cell reprogramming (Mosteiro et al., 2016). The higher expression of EMT-TF and reprogramming factors (RF) blocks some of the cell cycle regulators and modifies epigenetic marks and histones, and the appearance of stemness properties and the reprogramming of cells. The effect could be further reinforced by the fact that dysfunctional mitochondria induce the production of ROS, which are direct inducers of RF. Also, ROS-activated NF-kB contributes to the positive feedback loop originated by IL-6 stimulation.

Recently, it was shown that senescent keratinocytes, in addition to the classical hallmark features of senescence (senescence-associated β-Galactosidase (SA-βGal) activity, cell cycle arrest, p53, p16, p21, and Rb expression, ROS production, H3K9me3 marks and others) also showed increased expression of genes usually associated with CSC (i.e., CD34, Prom1, CD44, and Nestin, among others) (Ritschka et al., 2017). This gene expression up-regulation was independent of the senescence inducer, and the transient exposure to SASP was found to be responsible for this stem-like cell markers. The authors propose that in these conditions, stem-like cells that reside in a more plastic dynamic state might be more prone to transformation (Ritschka et al., 2017). Cellular stemness functions obtained by reprogramming factors and/or EMT-TF must be able to cross-talk with the different controllers of the cell-cycle. In fact, cellular differentiation and senescence are regulated by the activity of major cell cycle repressors, such as p53, Rb, p16, Arf, and p21, the expression of which is targeted by the reprogramming factors and EMT-TF (Strauss et al., 2011). The stem-like cell transcriptional program is activated by alterations of the epigenetic machinery that favor self-renewal and pluripotency (Poli et al., 2018b). Among these, DNA methylation (by DNA methyltransferases, DNMT) and de-methylation (by Ten-eleven translocation proteins, TET) and histone modifiers define the cellular transcriptional program. For example, IL-6 increased the methylation of p53 and p21 in A549 cancer cells by expression of DNMT1 (Liu et al., 2015). Also, hypermethylation of a negative regulator of Wnt signaling is an early event during colorectal tumorigenesis (Zhang et al., 2008). In breast cancer it has been shown that EZH2 hyperactivation is sufficient for malignant transformation and aggressiveness, suggesting that H3K27me3 marks are necessary for the gaining of CSC properties (increased invasion) (Kleer et al., 2003). Recently, a complex role of these cell cycle regulators in senescence or reprogramming was established; while p16 and Arf were necessary for senescence induction, IL-6 secretion and reprogramming, p53 and p21, known to represent a barrier for cell reprogramming, were dispensable (Mosteiro et al., 2018). Since ROS is produced during senescence and is critical for cell reprogramming (Zhou et al., 2016) and activation of NF-kB (Escobar et al., 2012) a positive feedback loop may be induced by IL-6 stimulation (Figure 3).

Since cell reprogramming proceeds through sequential steps due to transcriptional regulation of reprogramming factors in a certain order and with a particular intensity, the effect of the TME (SASP, for instance) will depend on the spatial and temporal relationship between stimuli and functional targets. In this sense, their short- or long-term induction defines the outcome of cells toward cellular senescence or cell reprogramming (Mosteiro et al., 2016). The number of senescent cells and therefore the quantity and composition of SASP defines not only which cells are likely to be reprogrammed but also with which intensity. The specific microenvironments in which the tumor cells reside and the contextual signals produced are the most important determinants defining their reprogramming capabilities and stemness functions.

For example, in an *in vitro* model of breast cancer, the IL-6/Stat3 axis has been shown to be critical in the conversion of non-CSC into CSC (Kim et al., 2013). Also, in an *in vivo* mouse model a positive correlation between senescence and cell reprogramming was found and it was shown that this effect was mediated by soluble factors (Mosteiro et al., 2016). The authors found that Nanog+ cells generally appear in close proximity to clusters of senescent cells in similar reprogrammable mice. The communication between senescence and cell reprogramming was enabled by the cytokine-rich microenvironment associated with senescent cells, in which IL-6 was identified as the critical soluble factor responsible for promoting de-differentiation (Mosteiro et al., 2016). Pharmacological inhibition of NF-kB, a TF activated by inflammatory cytokines (including IL-6, the most prominent cytokine of the SASP) diminishes significantly cell reprogramming. Also inhibition of its downstream kinase effector PIM1 has the same effect (Mosteiro et al., 2016). In the breast cancer cell lines T47D and MCF-7, the stimulation with IL-6 induces the expression of PIM1 and the expression of EMT and stemness markers (Gao et al., 2019). Here again, knockdown of PIM1 or inhibition of the mediator STAT3 abrogates stemness markers and function, while overexpression of PIM1 increases invasion and EMT/CSC markers (Gao et al., 2019).

Also, in the immortalized HEK cells model (Castro-Vega et al., 2013), HEK cells stimulated with a senescent-conditioned media (SCM) upregulated the reprogramming factors KLF4, NANOG, and OCT4 and formed efficiently spheres in low-binding conditions (Castro-Vega et al., 2015). In the MCF-7 cell line, SCM stimulation not only induces the EMT-TF but also the reprogramming factors OCT4 and KLF4 (Ortiz-Montero et al., 2017). The SCM-abundant IL-8 and IL-6 cytokines on their own also induced the expression of NANOG, OCT4, and SOX2 or KLF4, respectively. Here again, immune neutralization of these cytokine in the SCM reduces the expression of EMT-TF and some of the reprogramming factors (Ortiz-Montero et al., 2017). In the study by Mosteiro et al. (2016) it was also shown *in vitro*, that cell reprogramming efficiency was strongly enhanced by the SCM obtained from damaged cells and that IL-6 immunodepletion from the SCM abolished cell reprogramming. Using a cellular model of immortalization and oncogenic transformation it was shown that the homeobox transcriptional regulator SIX1 has a pro-tumorigenic action induced by the repression of a senescence-related signature, including p16Ink4a (Oliveras-Ferraros et al., 2012; De Lope et al., 2019). SIX1 overexpression was accompanied by an increase in SOX2 levels and activity and the induction of a de-differentiated tumor phenotype.

These results show that senescence, and more exactly, specific soluble factors within SASP, can induce the reprogramming of cancer cells, and that this de-differentiation process is responsible in part for the cellular plasticity of cancer cells and undoubtedly for the several complications observed during cancer progression and treatment. In fact, prominent SASP expression and senescent cells accumulation have been shown to be responsible for tumor progression and aggressiveness (Kang et al., 2011; Rodier and Campisi, 2011).

### Chemotherapy-Induced Senescence and Cell Reprogramming

Cancer stem cells can survive and are able to proliferate after chemotherapy (Singh et al., 2004; Visvader and Lindeman, 2008; Alison et al., 2011; Kreso and Dick, 2014) and it is well established that the mechanisms of this resistance are multiple (Phi et al., 2018). Since senescent processes can be induced as a consequence of uncontrolled cell growth by oncogene stimulation or anti-cancer drug treatments, senescence occurring in the TME and intrinsically in the tumor cells can impact importantly cancer progression. In fact, in multiple myeloma it has been shown that SASP produced by senescent cells within the tumor induce the emergence, after therapy-induced genotoxic stress, of CSC with the characteristic self-renewal and differentiation capacities, and with the ability to migrate toward chemokines released by the senescent non-CSC (Cahu et al., 2012). Also, tumor cells with defective apoptosis respond to chemotherapy by entering to a premature senescent state and, contrary to the traditional perception of the irreversibility of this process, some of these cells spontaneously revert to a proliferating cell phenotype in culture (Beauséjour et al., 2003). These experiments, could explain in part early relapses after termination of drug treatment (Yang et al., 2015). Additionally, it has been shown that cancer cells emerging from senescence after drug therapy increased the expression of CSC markers, CD34 and CD117 in lung cancer cells (Sabisz and Skladanowski, 2009), and CD133 and the reprogramming factor OCT4 in breast cancer cells (Achuthan et al., 2011). Importantly, emergence, maintenance and migration of senescent cancer cells were likewise molded by SASP (Cahu et al., 2012).

Reactivation of antioxidant enzymes and the subsequent low reactive oxygen species (ROS) levels were also a principal characteristic of the cells that emerge from senescence (Achuthan et al., 2011). This is a meaningful finding, since it has been shown that low ROS levels allow stem cells to survive in adverse conditions (Chen et al., 2006). Importantly the senescence revertants displayed a gene expression profile different from the parental cells and had also increased migration and invasion capabilities (Yang et al., 2017). The results showed that in 80% of the tumors, the revertant cells outgrew the parental cells. Intriguingly, a subset of senescence-activated genes remains active in the revertants (although to a lesser extend) (Yang et al., 2017), suggesting that differential long lasting senescent imprinting could be responsible for a non-genetic heterogeneity. Also, in experimental models of acute leukemia, cells emerging from senescence showed enhanced expression of stem cells markers, clonogenic growth and tumor-initiation potential (Milanovic et al., 2018). In the same study it was shown that senescence provokes the cell-autonomous reprogramming of non-stem tumor cells into *de novo* CSC, not only in hematological malignancies but also in cancer tissues of different origins. Intriguingly, emerging clones with different combination of activated genes could also show dynamic cell conversion adding a new element to cell plasticity (Milanovic et al., 2018). Moreover, differential cellular reprogramming may also occur in tumor cells that have undergone senescence reversal, contributing to tumor heterogeneity and aggressiveness.

As indicated above, modifications of the host TME by the different drug treatments available for cancer play also an important role by providing survival and protective niches for tumor cells. Accumulation of senescent cells could induce a SASP-dependent survival niche that can favor senescence scape of premalignant cells or the survival of adjacent clones with lower fitness, eventually inducing disease relapse (Guillon et al., 2019). This is another important element related to EMP, stemness, cell reprogramming and the cell plasticity developed and its connection with the acquired drug resistance (Singh and Settleman, 2010; Holohan et al., 2013; Jolly et al., 2015a). Though it is difficult to prove that cellular plasticity is responsible for drug-resistance recent lineage tracing experiments, transplantation studies and functional analysis have allowed finding indirect evidence of this (Le Magnen et al., 2018). For example, it was shown in advanced prostate cancer that drug resistance occurs by *trans*-differentiation of luminal adenocarcinoma into neuroendocrine-like cells (Zou et al., 2017). In non-small-cell lung tumors, resistant tumors with the original EGFR mutations show a phenotypic transition to small-cell neuroendocrine lung cancer suggesting a *trans*-differentiation process of the original adenocarcinoma (Oser et al., 2015). Also in other cancer models it has been shown that the plasticity of the hybrid E/M phenotypes not only favors collective migration, tumor initiation capabilities and metastatic potential, but also therapy resistance (Jolly et al., 2019). Cells that acquire a mesenchymal phenotype are in general resistant to drug treatment (Creighton et al., 2009; Oliveras-Ferraros et al., 2012; Shibue and Weinberg, 2017). The overexpression of EMT-TF in the luminal breast cancer cell line MCF-7 induced protection against the DNA-damaging agent doxorubicin (Kajita et al., 2004). In this work, the MCF-7 cells not only lost cell-cell contacts but also acquired invasive growth capabilities. A similar phenomenon was also reported for ovarian cancer (Haslehurst et al., 2012) and hepatocellular carcinoma (Dong et al., 2017). All these experiments demonstrate that cells that exhibit a high degree of cellular plasticity and stem-like cell functions are resistant to drug treatment.

In this sense, it has been suggested that blocking this cellular plasticity directly or by modulating the signals originated in the TME and responsible for this dynamic behavior could be important to increase susceptibility to therapy and control cancer progression (Yuan et al., 2019). Although the fundamental molecular mechanisms of drug resistance-induced cellular plasticity remain unsolved, it is believed that targeting the EMP pathways or the CSC maintenance programs, or particular E/M phenotypes with distinctive CSC attributes are thought to be valuable therapeutic strategies (Le Magnen et al., 2018). For example, the EMT phenotype can be manipulated by blocking the associated transcription factors (EMT-TF), inducing a reversal to an epithelial phenotype that in general is not linked to drug resistance in cancer cells (Li et al., 2009). Also relevant, prevention of CSC self-renewal function by inhibition of the PI3K/Akt/mTOR signaling axis may allow the evasion of chemotherapy resistance (Singh and Settleman, 2010).

Since cancer cell plasticity occurs by hijacking mechanisms present in the physiology of normal tissues, the pathways controlling it should be quite similar and susceptible to comparable blocking strategies. Cross-talk between CSC and the TME must be considered since during cancer progression they develop and share some characteristics that help functional adaptation of CSC, and therapeutic approaches must then target common cell plasticity properties of TME and CSC on one hand, but also some of the specificities found in the CSC.

## Cellular Senescence and Inflammation Propagation

In general it is believed that senescent cells comprise a small fraction of tissues (Wiley et al., 2017). Nevertheless, the fact that senescence is a phenomenon that can be propagated in the TME by different stimuli suggests that its relevance in cancer progression could be more important than thought before. Also, there is a close relationship between senescence and inflammation and their role in tumor progression when the producing stimuli are long-lasting (Lasry and Ben-Neriah, 2015). The fact SCM can induce the secretion of pro-inflammatory cytokines and that this in turn induces senescence imply that a double reinforcing loops can be established (Acosta et al., 2008; Kojima et al., 2013; Ortiz-Montero et al., 2017). This type of senescence/inflammation dissemination involves not only tumor cells but also other cells within the TME (Acosta et al., 2013).

The relevance and consequences of the presence (or not) of senescent cells in breast cancer is clearly exemplified with the MCF-7 and MDA-MB-231 breast cancer cell lines. The MCF-7 luminal cancer cell line has been classified as a senescent-cell progenitor subtype showing lower expression of the SA-βGal (Mumcuoglu et al., 2010). This cell line can differentiate into luminal and myoepithelial cell types and do not secret the pro-inflammatory cytokines IL-6 or IL-8. This is opposed to the basal/mesenchymal cell line MDA-MB-231, a highly IL-6 and IL-8 secreting cell line, classified as an immortalized-cell progenitor cell line that do not express SA-βGal (Mumcuoglu et al., 2010). Breast cancer cells exposed to the pro-inflammatory IL-6 cytokine exhibited enhanced invasion capacity and increased drug resistance (Dethlefsen et al., 2013); also, inflammatory microenvironments are known to expand the CSC pool and increase tumor initiation (Shenoy et al., 2012). Exposure of the MCF-7 cell line to SCM or to the pro-inflammatory cytokines IL-6 and IL-8 induced stem-like cell properties and aggressiveness attributes of an otherwise low aggressive cell line (Ortiz-Montero et al., 2017). Moreover, exposure to the SCM induces senescence propagation in MCF-7 cell cultures and remarkably the secretion of the cytokines IL-6 and IL-8 by this supposedly non-secreting IL-6 and IL-8 breast cancer cell line, a phenomenon that allows reinforcement of senescence by inflammatory cytokines potentiating cellular plasticity (Ortiz-Montero et al., 2017).

On the other hand, addition of IL-6 and IL-8 had no effect on the MDA-MB-231 breast cancer line. But, neutralization of the IL-6 and IL-8 cytokines affected slightly cell morphology and cell migration capacity and induced the expression of epithelial cell markers, suggesting that in this “pre-set” mesenchymal subtype, neutralization of pro-inflammatory cytokines affected differentially cell functions. Nevertheless, this breast cancer cell line presented abnormal differentiation to the osteoblastic lineage and it was suggested that its aggressiveness was more related to its abnormal differentiation-induced cell heterogeneity (Ortiz-Montero et al., 2017). Also, IL-8 has been associated with CSC and enhanced migration, invasion and metastasis in breast cancer (Freund et al., 2003; Charafe-Jauffret et al., 2009). During persistent inflammation, senescent cells accumulated and spread, and abundant signal responsible for cell damage and cell reprogramming contribute importantly to CSC plasticity (Mosteiro et al., 2016; Ortiz-Montero et al., 2017). Recently, it was shown that senescent cells are very heterogeneous in gene expression profiles (Wiley et al., 2017). One can imagine that surrounding cells will be affected in different ways depending on this cell heterogeneity.

*In vivo*, the situation should be more complicated and it would be very difficult to know how senescent cells evolve in TME with an immune system (IS) that could be partially effective in the beginning of the anti-tumoural immune response, but functionally impaired latter. Also, the appearance and maintenance of senescent cells would be influenced by the genetic instability induced by the continuous genotoxic stress, the rate at which senescence propagates in the TME and the persistence of inflammatory events. All these phenomena may also have important consequences on the robustness of the IS response, with serious consequences in the long-term. In this sense, the growth arrest observed by imagine techniques and interpreted as effective cytostatic responses will not always indicate a favorable response (Guillon et al., 2019), specially, if this guarantees the spread of senescence and the appearance of more aggressive CSC.

The IS also affect the TME in different ways; in particular it can contribute to an increased stress and to the accumulation of senescent cells. SASP of senescent cells facilitates leukocytes recruitment through various chemokines (Mantovani, 2004). Although in principle this would allow clearance of pre-malignant cells by the innate and/or adaptive IS, secretion of cytokines by immune cells (macrophages, neutrophils, myeloid-derived suppressors cells (MDSC), NK cells and lymphocytes) and their accumulation also affect significantly the TME. In particular, TGF-β secreted by TAM plays an important role. Although its effect has been shown to be highly context-dependent (Wahl, 2007), its secretion by macrophage or TAM induces senescence in primary and tumor cells (Katakura et al., 1999; Senturk et al., 2010). Also IL-6, secreted by tumor cells, infiltrating lymphocytes, TAM, MDSC and other myeloid cells, induces senescence and SASP dissemination in the TME (Coppé et al., 2010; Salminen et al., 2018). The immunosuppression induced by MDSC, through regulatory T-cells, impairs the clearance of senescent and cancer cells. IL-8, secreted by macrophages, epithelial cells, CAF and other infiltrating immune cells, induces senescence and promote recruitment of additional leucocytes, neutrophils and MDSC (Ginestier et al., 2010).

Additionally, SASP may also favor an immunosuppressive TME (Toso et al., 2014), resulting in an impaired immune surveillance, malfunctioning of immune cells and hence carcinoma progression (Kang et al., 2011). Since senescence may appear with different intensities (percentage of senescent cells or the presence of cells in different stages of senescence), and in different cell contexts (genetic or functional), its impact in the IS, via diverse SASP compositions, could change in the course of the disease. Of note, an impaired functionality of the IS with secretion of pro-inflammatory mediators (even in reduced amounts) in addition to SASP, contributes to the accumulation of senescent cells and the expansion of senescence due to a reduced cell clearance and the cytokine paracrine effect on the TME and also on immune cells (Prieto and Baker, 2019). In addition to causing the accumulation of senescent cells, SASP also allow the EMP, the cell reprogramming of tumor cells with the appearance of plastic CSC in a TME in which interactions between the different cells determine tumor growth and disease progression (Demaria et al., 2017; Milanovic et al., 2018).

### Cooperation Between Different CSC Within a Senescent TME

As mentioned, SASP are responsible for spreading senescence in tumor cells and adjacent cells by a paracrine mechanism (Baker et al., 2011; Acosta et al., 2013; Campisi, 2013). In this manner, cells within the TME may display different levels of senescence and diverse stemness-associated features. Permanent fuelling with SASP would eventually allow cancer cells to acquire the ideal combination of stemness features with particular normal cell traits able to produce cells with special attributes responsible for increased aggressiveness. Gene expression signatures in senescent cells differ partially within the cell subpopulation present in TME (Wiley et al., 2017) and therefore one can imagine that their soluble secreted factors would have differential influence in the behavior of the tumor and surrounding cells. As already mentioned, CSC cultured in a conditioned medium obtained from senescent non-CSC remain in an undifferentiated state and develop enhanced migration capacity toward the same conditioned medium (Cahu et al., 2012), the latter event being a good example of cooperation between non-CSC and CSC. CSC with increased migration capacity would eventually migrate to new niche were SASP effect is reduced and could eventually differentiate into non-CSC rebuilding the bulk of tumor cells. The communication between the different cells present in the TME can be produced by protein matrix connections or soluble factors secretion from the different clones present and might produce benefit for all the participant cells, a sort of mutualism (Axelrod et al., 2006).

In a mouse model of breast cancer it was shown that interclonal cooperation is essential for the maintenance of tumors (Cleary et al., 2014). Abnormal secretion of signaling molecules generates tumors composed of basal and luminal cell subtypes. In conditions in which Wnt was withdrawn, basal subclones were able to recruit Wnt-secreting luminal subclones to restore tumor growth (Cleary et al., 2014). In a colorectal cancer model, it was shown that CSC-like cells and chemoresistant cells could confer chemoresistance on the surrounding naïve cancer cells (Bose et al., 2011). It was shown that this was mediated by soluble factors through the activation of growth and survival signaling pathways. In the immortalized HEK model, we have shown that explanted cells from tumors, that had acquired an autonomous tumorigenic capability (independent of the presence of senescent cells) expressed both epithelial and mesenchymal markers (hybrid E/M phenotype), but with variable expression of CD24. When these cells were tested for tumourigenicity, it was shown that those having increased stemness functions (CD24+) were not tumourigenic, while the CD24 negative cell population, having reduced stemness functions, was as tumourigenic as the parental cell line (Ortiz-Montero et al., 2018) and presented homogeneous tumors with epithelioid morphology. Tumors formed by the simultaneous inoculation of a mixture of the non-tumourigenic CD24+ and the tumourigenic CD24 negative cell populations, were more heterogeneous with epithelioid and fibroblastoid cells and the presence of mesenchymal markers (alfa-SMA and vimentin). This suggests that non-tumourigenic cells may influence the differentiation of the tumors and contribute to cell heterogeneity. Although the mechanisms by which the presence of CD24+ cells contributes to cell heterogeneity within the tumor is unknown, it is interesting to appreciate that the CD24+ cell population has a more pro-inflammatory SASP. In fact both IL-6 and IL-8 were increased in a SCM obtained from CD24+ cells; this in turn could influence the CD24 negative population and clearly the TME (Ortiz-Montero et al., 2018).

These studies showed that phenotypically or genotypically distinct cell clones interact to the benefit of one or more clones within the tumor, contributing to heterogeneity and CSC plasticity (Neelakantan et al., 2015). All these cooperative processes should be very relevant also during the generation of metastatic clones or during the spread of the existing ones (Neelakantan et al., 2015). Intriguingly, tumor collapse can occur if the driver subclone gets outcompeted by the fast growing and less-fit dependent subclone (Marusyk et al., 2014). Mathematical modeling suggested that non-cell autonomous driving, together with clonal interference, stabilizes sub-clonal heterogeneity and enhancing inter-clonal interactions and the appearance of new phenotypic traits (Marusyk et al., 2014). This proposal is very relevant when its come to interpreting cancer genomic data. Some mutations detected at low allelic fractions and believed to represent late events in tumor progression may instead denote early events that allow interclonal cooperation and disease evolution (Cleary et al., 2014).

## Conclusion

Phenotypic intratumour heterogeneity in cancer cells limits treatment against this disease. Cellular plasticity of cancer cells is a very complex process that can be induced by different mechanisms (EMP, de-and *trans*-differentiation processes, cell reprogramming, for example) and can be molded by the diverse stimuli present in the TME. In particular, the senescence and inflammation phenomena frequently occurring during cancer development and progression must have a very important role. Since this TME evolve permanently based on the incoming cells and the propagation of senescence and the composition of SASP, its effect is variable in the course of the disease. By this mean, cells are able to transit between different phenotypic states having different stemness properties and functions. Cancer cells with this dynamic behavior are more aggressive since they are more plastic and can respond/adapt more efficiently or faster to the diverse hurdles established during the different stages of carcinogenesis. These cells acquire/use particular functional properties, according to their needs, allowing them for example to proliferate, colonize a particular tissue and form a tumor; alternatively, these plastic cells may have other stem-associated features that allow them for example to migrate and invade new tissues; also they could be more prone to a cell cooperative behavior.

The acquisition of a specific set of stem cell features to overcome physical/physiological barriers may be sufficient or even more effective than to acquire a full-blown stemness program. Different stem-like cell properties would confer diverse cell advantages useful in the different stages of cancer progression. These features would be differentially influenced not only by the cell context (differentiation status, lineage background, etc.), the environmental cues (presence of inflammatory and senescent soluble factors) and immune status, but also, by radio- or chemotherapy. Intriguingly, they can be harbor by different subclones that cooperate to enhance they migration, colonization, growth, self-renewal and/or differentiation capabilities.

Stem cell functions (self-renewal, clonogenicity, multilineage differentiation capacity, migration, invasion, etc.) if adequately quantified and categorized according to the stage of cancer progression could give quantitative information about the adaptability of the tumor cell, and hence about CSC aggressiveness and tumor formation capacity. The phenomenon of cellular plasticity induced by a senescence-associated inflammatory milieu increases the difficulty of developing a suitable cancer therapy, and may explain the poor overall survival despite initial favorable responses to treatment. Blocking inflammation (Harrison et al., 2007; Loberg et al., 2007) and/or senescence (Myrianthopoulos et al., 2019; Zhang et al., 2019), or inhibiting the reversal of senescence in tumor cells (Yang et al., 2017), may reduce development, progression, and recurrence of cancer.

## Author Contributions

JPV selected the topic, performed the revision and wrote the manuscript.

## Conflict of Interest

The author declares that the research was conducted in the absence of any commercial or financial relationships that could be construed as a potential conflict of interest.
